# Comparison of conventional BLUP and single-step genomic BLUP evaluations for yearling weight and carcass traits in Hanwoo beef cattle using single trait and multi-trait models

**DOI:** 10.1371/journal.pone.0223352

**Published:** 2019-10-14

**Authors:** Hossein Mehrban, Deuk Hwan Lee, Masoumeh Naserkheil, Mohammad Hossein Moradi, Noelia Ibáñez-Escriche

**Affiliations:** 1 Department of Animal Sciences, Shahrekord University, Shahrekord, Iran; 2 Department of Animal Life and Environment Sciences, Hankyong National University, Jungang-ro 327, Anseong-si, Gyeonggi-do, Korea; 3 Department of Animal Sciences, University College of Agriculture and Natural Resources, University of Tehran, Karaj, Iran; 4 Department of Animal Sciences, Faculty of Agriculture and Natural Resources, Arak University, Arak, Iran; 5 Institute for Animal Science and Technology, Universitat Politècnica de València, València, Spain; Universidade Federal de Mato Grosso do Sul, BRAZIL

## Abstract

Hanwoo, an important indigenous and popular breed of beef cattle in Korea, shows rapid growth and has high meat quality. Its yearling weight (YW) and carcass traits (backfat thickness, carcass weight- CW, eye muscle area, and marbling score) are economically important for selection of young and proven bulls. However, measuring carcass traits is difficult and expensive, and can only be performed postmortem. Genomic selection has become an appealing procedure for genetic evaluation of these traits (by inclusion of the genomic data) along with the possibility of multi-trait analysis. The aim of this study was to compare conventional best linear unbiased prediction (BLUP) and single-step genomic BLUP (ssGBLUP) methods, using both single-trait (ST-BLUP, ST-ssGBLUP) and multi-trait (MT-BLUP, MT-ssGBLUP) models to investigate the improvement of breeding-value accuracy for carcass traits and YW. The data comprised of 15,279 phenotypic records for YW and 5,824 records for carcass traits, and 1,541 genotyped animals for 34,479 single-nucleotide polymorphisms. Accuracy for each trait and model was estimated only for genotyped animals by five-fold cross-validation. ssGBLUP models (ST-ssGBLUP and MT-ssGBLUP) showed ~19% and ~36% greater accuracy than conventional BLUP models (ST-BLUP and MT-BLUP) for YW and carcass traits, respectively. Within ssGBLUP models, the accuracy of the genomically estimated breeding value for CW increased (19%) when ST-ssGBLUP was replaced with the MT-ssGBLUP model, as the inclusion of YW in the analysis led to a strong genetic correlation with CW (0.76). For backfat thickness, eye muscle area, and marbling score, ST- and MT-ssGBLUP models yielded similar accuracy. Thus, combining pedigree and genomic data via the ssGBLUP model may be a promising way to ensure acceptable accuracy of predictions, especially among young animals, for ongoing Hanwoo cattle breeding programs. MT-ssGBLUP is highly recommended when phenotypic records are limited for one of the two highly correlated genetic traits.

## Introduction

Hanwoo is a breed of beef cattle indigenous to Korea, very popular due to its high meat quality. The excessive marbling found in this breed has become an important factor influencing the quality of meat products in commercial beef markets [1, 2]. Marbled Hanwoo meat is considered to be a healthy food product, as it contains a large amount of monounsaturated fatty acids, which are known to reduce low-density lipoprotein cholesterol levels in blood, while increasing high-density lipoprotein cholesterol levels [3].

The Hanwoo beef industry mainly aims to increase both the quantity (yearling weight—YW, backfat thickness—BT, carcass weight—CW, and eye muscle area—EMA) and quality (marbling score—MS) of meat. At present, Hanwoo cattle are selected by conventional methods involving two tests: a performance test for young bulls followed by a progeny test for selected young bulls. The estimated breeding values (EBVs) for MS and YW obtained from the classical animal model are used for selecting young bulls, while the EBVs for BT, CW, EMA, and MS are used for selecting proven bulls [4]. However, these conventional breeding methods are not very accurate or efficient, and have a large generation interval. Measurement of traits such as meat quality and quantity is difficult, and can only be performed when an animal reaches maturity, thereby delaying the verification of breeding results [5].

Advances in genome-wide single-nucleotide polymorphism (SNP) panels have overcome disadvantages of conventional breeding methods. SNP panels enable accurate and cost- and time-effective prediction of genomically estimated breeding values (GEBVs), even in young animals. Studies on beef cattle have shown that several quantitative traits are polygenic [6–8]. The accuracy of genomic selection for these traits can be increased by improving the estimation of correlations using a genomic relationship matrix [9]. Previously, the most common methods for genomic predictions using SNP panels involved a multistep approach that used only the phenotypic records associated with genotyped individuals. These methods require estimation of weights for SNP effects, mostly using Bayesian procedures [10, 11]. However, the accuracy of these methods is not optimal [9] because phenotypic information from relatives cannot be used. In addition, these methods have several drawbacks such as bias and loss of information in animals with few progenies, as well as operational complexity [12]. Misztal et al. [13] have proposed a method known as single-step genomic best linear unbiased prediction (ssGBLUP), where genotyped and nongenotyped individuals can be evaluated simultaneously. In ssGBLUP, the pedigree-based relationship matrix (**A**) and relationship matrix based on genomic information (**G**) are combined into a single matrix, **H** [13, 14]. The inverse of **H** has a simple form and can substitute for the inverse of **A** in existing equations [15]. This method is simpler, applicable to complicated models, and generally as accurate as multistep methods [16]. In addition, ssGBLUP can take selection bias into account, when selection is based only on genotypes [17].

Numerous genomic selection studies have demonstrated the advantages of ssGBLUP in various domesticated species, including beef cattle [18–20], dairy cattle [17, 21], pigs [22, 23], and chickens [24]. Most of these studies have focused on single-trait analyses, however, many traits, such as YW and carcass traits, are genetically correlated in beef cattle [1, 5]. In simulated [25] and real data [26] on quantitative traits controlled by different genetic architectures, higher prediction accuracy has been demonstrated for a multi-trait genomic model than for a single-trait genomic model. However, the performance of genomic prediction for YW and carcass traits in Hanwoo beef cattle has not yet been evaluated by means of a multi-trait genomic model.

This study was aimed at evaluating the accuracy of breeding values for economically important carcass traits and YW in Hanwoo beef cattle, obtained using four models: conventional single-trait BLUP (ST-BLUP), multi-trait BLUP (MT-BLUP), single-trait single-step genomic BLUP (ST-ssGBLUP), and multi-trait single-step genomic BLUP (MT-ssGBLUP). We found that combining pedigree and genomic data in the ssGBLUP model may be a promising way to ensure acceptable accuracy of predictions, especially among young animals, for ongoing Hanwoo cattle breeding programs.

## Materials and methods

### Ethics statement

DNA samples were obtained from semen and/or blood samples collected by veterinarians. Ethics committee approval was not required for semen and blood collection, as they were collected specifically for this study from the Hanwoo Improvement Center(HIC) of the National Agricultural Cooperative Federation, which was involved as a partner in this research project that was supported by a grant from the IPET Program (No. 20093068), Ministry of Agriculture, Food and Rural Affairs, Republic of Korea. Pedigree data were recorded by the Korean Animal Registration Association (http://www.aiak.or.kr), data for growth traits were obtained from the Hanwoo Improvement Center (http://www.limc.co.kr), and data for carcass traits were recorded by special inspectors, from the Institute of Korean Animal Products Evaluation (http://www.ekape.or.kr), at the slaughterhouse. Pedigree and phenotypic data related to growth and carcass traits were generated following the protocol for progeny test program, as notified by the Ministry of Agriculture, Food and Rural Affairs based on livestock law in Korea. HIC, as an enforcement institution for the testing program for selecting proven Hanwoo bulls, is obligated to maintain data and ownership of enrolled animals under notice.

### Phenotypic and pedigree data

The dataset consisted of YW phenotypes of 15,279 purebred Hanwoo cattle (8,966 bulls and 6,313 steers) and carcass traits of 5,824 steers (Table 1).

**Table 1:** Summary statistics for phenotypic data used to estimate variance components in Hanwoo cattle.

Trait (units)	Number of animals with record (and genotype)	Mean (SE)	Min.	Max.	SD	**CV%**
BT (mm)	5,824 (1,151)	8.71 (0.05)	1.00	30.00	3.71	42.61
CW (kg)	5,824 (1,151)	343.96 (0.60)	158.00	519.00	45.61	13.26
EMA (cm^2^)	5,821 (1,151)	78.90 (0.12)	40.00	123.00	9.12	11.56
MS (score)	3,991 (1,151)	3.33 (0.03)	1.00	9.00	1.61	48.46
YW (kg)	15,279 (1,541)	342.06 (0.38)	133.86	535.90	47.48	13.88

BT, backfat thickness; CW, carcass weight; EMA, eye muscle area; MS, marbling score; YW, yearling weight.

The animals analyzed in this study were born between 1989 and 2015. Pedigree data of 50,115 animals, obtained on tracing the pedigree file back up to 11 generations, were used in the animal model. YW for each animal was estimated from weight at termination (t) of the test (body weight at ~365 days, W_t_) and pervious weight at time (t _-1_) before termination (body weight at ~180 days, W_t − 1_), according to Park et al. [5], as follows:

$$YW=\left[(\frac{W_{t}-W_{t_{-1}}}{t-t_{-1}})\times (365-t_{-1})\right]+W_{t_{-1}}$$

MS was assessed using a categorical system of nine classes, ranging from the lowest score of one (no marbling) to the highest score of nine (abundant marbling). The records on MS collected before 2005 were excluded as they were not consistent with the newly adopted 9-point MS system. Carcass traits were measured in ~24-month-old steers by ribbing between the 13^th^ rib and 1^st^ lumbar vertebra 24 h postmortem following the Korean carcass grading procedure, according to notification No. 2014–4 of the Ministry of Agriculture, Food and Rural Affairs.

### Genotypes

A total of 1,679 animals were genotyped using Illumina BovineSNP50K (n = 959) and HD 777K (n = 720) BeadChip (Illumina Inc., San Diego, CA, USA). The SNPs common to the 50K and 777K SNP chips were matched to increase the sample size, resulting in 45,304 SNPs. Animals with more than 10% of missing genotype data (73 heads of cattle) and without a phenotype for five traits of interest (n = 15) as well as animals with parent−progeny conflicts (n = 11) or deviation errors between the pedigree and genomic relations (n = 39) were excluded from the final analyses. SNPs with unknown positions (302 SNPs) and those located on sex chromosomes (1,150 SNPs) were removed from the analyses after quality control. Furthermore, SNPs with call rates lower than 0.98 (2,677 SNPs), minor allele frequencies lower than 0.01 (6,684 SNPs), and a Mendelian error (12 SNPs) were also excluded. The missing genotypes were imputed in the BEAGLE software [27]. Finally, genotypes for 34,479 SNP markers from 1,541 animals (386 bulls and 1,155 steers) were available. All genotyped animals had records for YW, while four genotyped steers lacked records for carcass traits.

### Statistical analyses

#### Estimation of variance components and corrected phenotypes

The variance components for YW and carcass traits were estimated based on the classical Bayesian multi-trait animal model (with pedigree and phenotype data), similar to Choi et al. [1] and Park et al. [5], as follows:

y=Xb+Zu+e (1)

where **y** is the vector of observations for the trait of interest; **b** is the vector of fixed effects, including batch-test place-sex (163 levels) and birth place (108 levels) for YW, batch-test place-slaughter date (391 levels, 201 levels for MS), birth place (86 levels, 76 levels for MS), and slaughter age (days from birth to slaughter) was considered as covariates for carcass traits (except BT, as there is no significant effect of slaughter age); **u** is the vector of random genetic additive effects; **e** is the vector of random residual effects; **X** and **Z** are incidence matrices related to fixed and random genetic additive effects, respectively. Var (**u**) = **G**⊗**A** and Var(**e**) = **R**⊗**I** were assumed, where **A** is the numerator relationship matrix, **I** is the identity matrix, and **G** and **R** are additive genetic and residual covariance, respectively, for the five traits. Priors for fixed effects (**b**) were uniform, and Gaussian distribution was assumed for random genetic additive effects (**u**), and inverse Wishart distribution was employed for variance components (**G** and **R**).

The model was in the gibbs2F90 software [28], and the Markov chain Monte Carlo process was run for 1,100,000 cycles with 100,000 iterations as burn-in with a thinning interval of 50. Convergence of the chain was checked by visual inspection of trace plots. The variance components and correlations were estimated as a posterior mean of 20,000 samples.

Furthermore, a single-trait animal model was used for comparison with the multi-trait model, assuming zero genetic and residual covariance among the traits.

The corrected phenotype for each trait and animal (**y**_c_) was calculated as the sum of the EBVs, and the residual (i.e., $\hat{u}+e$) was obtained from the multi-trait traditional animal model, as described by Onogi et al. [18] and Lee et al. [20].

### Models

#### Traditional evaluation (BLUP)

The multi-trait pedigree-based evaluations (MT-BLUP) were performed using the following animal model:

$$y_{c}=1\mu +Zu+e$$ (2)

where $y_{c_{i}}$ is the vector of observations for the *i*^th^ trait corrected for fixed effects; **1** is a vector of ones; μ is the overall mean; other notations are the same as in model Eq (1).

Furthermore, genetic and residual covariances for the five traits were assumed to be zero for the single-trait pedigree-based model (ST-BLUP).

#### Genomic evaluation (ssGBLUP)

In the ssGBLUP method, the statistical model was the same as that for the traditional evaluation (ST-ssGBLUP and MT-ssGBLUP referring to single- and multi-trait single-step genomic evaluations, respectively) except for the inverse of numerator relationship matrix **A**−^1^, which was replaced by matrix **H**−^1^ [13, 14]. This matrix was obtained in the preGSf90 software, as follows [15, 29]:

$$H^{-1}=A^{-1}+\left[\begin{array}{cc} 0 & 0\\ 0 & {\left(\alpha G+\beta A_{22}\right)}^{-1}-A_{22}^{-1} \end{array}\right]$$ (3)

where **A** is the numerator relationship matrix; **G** is the genomic relationship matrix; α (0.95) and β (0.05) are mixing parameters used to combine matrices **G** and **A**_22_ (the numerator relationship matrix for genotyped animals) to avoid singularity problems [30].

Variance components used in models MT-BLUP (ST-BLUP) and MT-ssGBLUP (ST-ssGBLUP) were estimated using the pedigree-based [20] multitrait (single-trait) animal model (Eq 1). Breeding values in all the models were obtained by means of the BLUPF90 software [28].

### Validation

Genotyped animals (1,541) were allocated to five mutually exclusive subsets for cross-validation by K-means clustering [31] based on pedigree relationship coefficients. The numbers of animals in the five groups of subsets for carcass traits (and YW) were 297 (367), 276 (357), 145 (180), 307 (468), and 126 (169), respectively. Of the five subsets, four served as training data, and the remaining subset was regarded as validation data; hence, each subset was predicted once from other subsets. The correlation between EBV / GEBV and corrected phenotypes of all animals in the validation population was defined as the predictive ability of EBVs / GEBVs for each fold number. The accuracy of each trait was estimated by dividing the predictive ability from the validation population data by the heritability root of the trait estimated by the classic multi-trait animal model (Eq 1). Accuracy was determined as the mean of accuracies for five-fold cross-validation procedures. The regression coefficient of the corrected phenotypes on EBV / GEBV was calculated as a metric of bias. The impact of additional phenotypic records of YW, compared with CW, on accuracy was investigated by analyzing equal numbers of animals for YW and CW as a training population (nongenotyped and genotyped animals with phenotypic records), while, the validation population size (genotyped animals without any phenotypic records) was kept constant in all analyses, according to Mehrban et al. [8].

## Results

### Estimation of variance components and correlations

Estimates of variance components and heritability for YW and carcass traits, except CW, were almost similar in the single- and multi-trait analyses. For CW, slight difference in environmental variances was noted between two models, but genetic variance was significantly increased (~45%) in the multi-trait model, further increasing heritability (~26%), when compared with the single-trait model. Regardless of the model chosen, the lowest and highest heritability corresponded to YW and MS, respectively (Table 2).

**Table 2:** Variance components and heritability (95% HPD) estimated from pedigree and phenotypic information in Hanwoo population.

		BT		CW		EMA		MS		YW	
		ST	MT	ST	MT	ST	MT	ST	MT	ST	MT
$\sigma_{a}^{2}$	**Mean**	**5.50**	**5.66**	**316.69**	**460.32**	**27.44**	**29.38**	**1.51**	**1.59**	**277.70**	**273.81**
	**Lower 95**	4.44	4.48	231.40	360.40	21.44	23.22	1.19	1.24	225.00	221.50
	**Upper 95**	6.67	6.82	406.40	562.30	33.80	36.12	1.83	1.96	332.40	327.20
$\sigma_{e}^{2}$	**Mean**	**5.58**	**5.54**	**710.65**	**721.79**	**35.15**	**35.52**	**0.97**	**0.91**	**758.85**	**761.44**
	**Lower 95**	4.70	4.60	632.10	640.50	30.00	30.15	0.72	0.63	716.60	718.70
	**Upper 95**	6.48	6.46	784.10	805.20	40.01	40.62	1.22	1.18	801.20	803.80
$\sigma_{p}^{2}$	**Mean**	**11.08**	**11.20**	**1027.34**	**1182.11**	**62.59**	**64.90**	**2.48**	**2.50**	**1036.54**	**1035.25**
	**Lower 95**	10.62	10.71	985.90	1132.40	59.97	62.06	2.35	2.37	1009.00	1007.60
	**Upper 95**	11.58	11.70	1069.30	1233.10	65.32	67.76	2.61	2.64	1064.10	1062.20
***h***^**2**^	**Mean**	**0.50**	**0.51**	**0.31**	**0.39**	**0.44**	**0.45**	**0.61**	**0.64**	**0.27**	**0.26**
	**Lower 95**	0.41	0.41	0.23	0.31	0.35	0.37	0.50	0.52	0.22	0.22
	**Upper 95**	0.58	0.60	0.39	0.47	0.53	0.55	0.72	0.76	0.32	0.31

BT, backfat thickness; CW, carcass weight; EMA, eye muscle area; MS, marbling score; YW, yearling weight. ST, MT, $\sigma_{a}^{2},\;\sigma_{e}^{2},\;\sigma_{p}^{2}$, *h*^2^ and HPD: single-trait analysis, multi-trait analysis, additive genetic variance, error variance, phenotypic variance, heritability, and the highest posterior density, respectively.

Genetic and phenotypic correlations among traits are presented in Table 3. According to the 95% highest posterior density (HPD), a significant genetic correlation was found for YW with CW (0.68, 0.84) and EMA (0.20, 0.49). Furthermore, a positive and significant genetic correlation was noted between CW and EMA (0.45, 0.67), between CW and MS (0.005, 0.32), and between EMA and MS (0.14, 0.44). Genetic correlations of BT with CW, EMA, MS, and YW and genetic correlation between MS and YW were not significant (Table 3).

**Table 3:** Estimates of genetic (upper diagonal) and phenotypic (lower diagonal) correlations between traits in Hanwoo population.

Traits	BT	CW	EMA	MS	YW
BT	1	0.14 (-0.01, 0.29)	-0.14 (-0.29, 0.01)	-0.06 (-0.22, 0.10)	-0.01 (-0.17, 0.15)
CW	0.28 (0.25, 0.31)	1	0.56 (0.45, 0.67)	0.17 (0.005, 0.32)	0.76 (0.68, 0.84)
EMA	0.01 (-0.02, 0.05)	0.57 (0.54, 0.59)	1	0.29 (0.14, 0.44)	0.34 (0.20, 0.49)
MS	0.07 (0.03, 0.10)	0.10 (0.07, 0.14)	0.21 (0.17, 0.25)	1	-0.16 (-0.33, 0.004)
YW	0.18 (0.15, 0.21)	0.70 (0.69, 0.72)	0.36 (0.33, 0.39)	0.01 (-0.03, 0.05)	1

BT, backfat thickness; CW, carcass weight; EMA, eye muscle area; MS, marbling score; YW, yearling weight. Numbers in parentheses are lower and upper 95% highest posterior densities.

### Model comparisons

**Fig 1:**
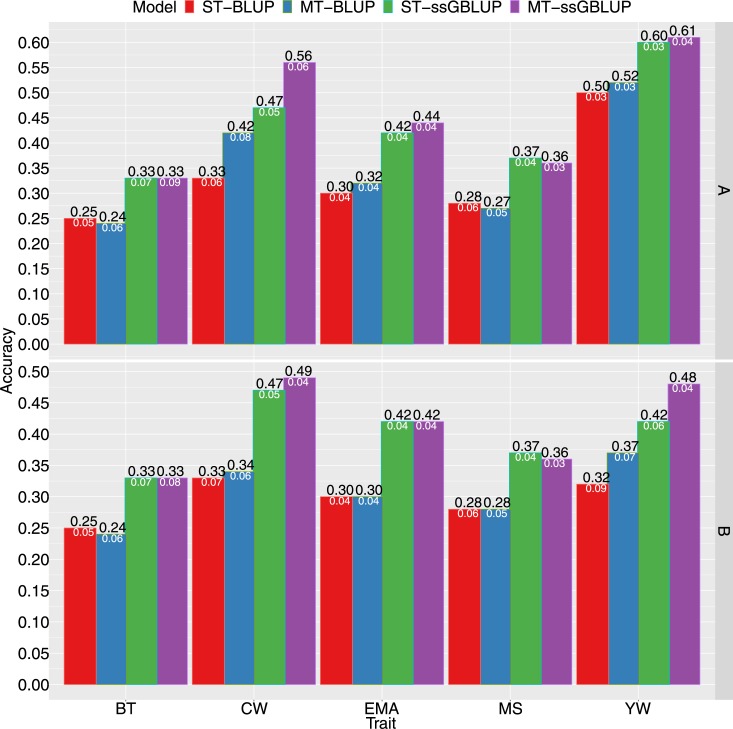
Accuracy of breeding values obtained using BLUP (ST-BLUP, MT-BLUP) and ssGBLUP (ST-ssGBLUP, MT-ssGBLUP) models. The means and standard errors for backfat thickness (BT), carcass weight (CW), eye muscle area (EMA), marbling score (MS), and yearling weight (YW) obtained using complete phenotypic data (A) and a reduced dataset for YW only (B) in Hanwoo population. The white numbers represent standard error (SE). ST and MT are single-trait and multi-trait analyses, respectively.

The mean realized accuracy of EBVs (ST-BLUP and MT-BLUP) was 0.25 for BT, 0.38 for CW, 0.31 for EMA, 0.28 for MS, and 0.51 for YW (Fig 1A and S1 Table). The accuracy of GEBVs for each trait was higher in the ssGBLUP model, compared to the BLUP model. The ssGBLUP model was more accurate than the BLUP model by approximately 35% for BT, 37% for CW, 39% for EMA, 33% for MS, and by 19% for YW, averaging at 31% more accuracy than BLUP. Relevant differences in the accuracy of single- and multi-trait models were not observed for the traits studied, except CW. Accuracy for multi-trait models MT-BLUP (0.42) and MT-ssGBLUP (0.56) was ~0.09 greater than that of single-trait models ST-BLUP (0.33) and ST-ssGBLUP (0.47). On the other hand, the gain in accuracy for MT-BLUP and MT-ssGBLUP models was 27% and 19% higher than that of ST-BLUP and ST-ssGBLUP models, respectively (Fig 1A).

For all traits, the regression coefficients ranged from 0.72 to 1.14 for single-trait BLUP, 0.68 to 1.18 for multi-trait BLUP, 0.81 to 1.32 for single-trait ssGBLUP, and 0.77 to 1.20 for multi-trait ssGBLUP methods (Table 4 and S2 Table).

**Table 4:** Regression coefficients of the adjusted phenotype on the EBVs and GEBVs in BLUP by single- and multi-trait (ST-BLUP and MT-BLUP) and ssGBLUP by single- and multi-trait (ST-ssGBLUP and MT-ssGBLUP) models in Hanwoo population.

Trait	ST-BLUP	MT-BLUP	ST-ssGBLUP	MT-ssGBLUP
BT	0.72 (0.15)	0.68 (0.16)	0.81 (0.18)	0.77 (0.20)
CW	1.09 (0.22)	1.07 (0.18)	1.32 (0.11)	1.19 (0.09)
EMA	0.84 (0.10)	0.92 (0.09)	1.05 (0.10)	1.05 (0.11)
MS	0.75 (0.14)	0.74 (0.13)	0.86 (0.09)	0.83 (0.08)
YW	1.14 (0.10)	1.18 (0.11)	1.18 (0.08)	1.20 (0.09)

BT, backfat thickness; CW, carcass weight; EMA, eye muscle area; MS, marbling score; YW, yearling weight. Numbers in parentheses represent standard error (SE).

In general, no obvious changes in regression of the adjusted phenotype on EBVs / GEBVs were observed between single- and multi-trait analyses for either BLUP or ssGBLUP models, regarding BT, EMA, MS, and YW. However, the bias was decreased when the MT-ssGBLUP model was used for CW instead of the ST-ssGBLUP model. The absolute deviation of regression coefficients from 1.0 was 0.32 and 0.19 for ST-ssGBLUP and MT-ssGBLUP, respectively. In terms of bias, the range of variations for carcass traits was in agreement with that reported by Lee et al. [20].

The phenotypic data on YW were considerably more abundant than those for carcass traits. Thus, the accuracy of carcass traits could be affected by additional phenotypic records because of the genetic correlation between YW and carcass traits obtained using multi-trait models. Therefore, we performed the analysis after reducing the YW data of the training population to the same size as CW data. No significant changes were revealed in mean realized accuracy of EBVs / GEBVs for BT, MS, and EMA, when compared with those obtained using the complete data. However, accuracy decreased for YW in all models, and for CW in MT-BLUP and MT-ssGBLUP (Fig 1A, Fig 1B and S3 Table). The smallest decrease in accuracy (21%) for YW was seen in the MT-ssGBLUP model, followed by models MT-BLUP and ST-ssGBLUP, with a decrease of 29% and 30%, respectively. The ST-BLUP model showed the largest reduction in accuracy (36%) for YW. For CW, this loss of accuracy was smaller, with a decrease of 12.5% in MT-ssGBLUP and 19% in the MT-BLUP model.

## Discussion

During the national genetic evaluation of Hanwoo, YW is one of the traits used at the performance test stage for selecting young bulls. Thus, YW is recorded for all candidate young bulls, but carcass traits are measured only for the offspring of selected young bulls as steers [4]. Hence, the analyses of carcass traits excluding the YW data would be biased, which can, however, be eliminated by a multi-trait analysis involving both YW and carcass traits [32]. Although the magnitudes of heritability estimated for BT, EMA, MS, and YW were similar to those reported previously for Hanwoo beef cattle with either single- or multi-trait models [1, 5, 33], CW showed a higher heritability with the multi-trait model. This could be due to the larger number of YW phenotypes included in this study in comparison previous studies [1, 5, 33], allowing the utilization of the high genetic correlation (0.76) between CW and YW. It must be noted that the genetic correlations among traits included in this study are in agreement with those reported by Choi et al. [1]. Onogi et al. [18] reported a heritability of 0.56 for CW in Japanese Black beef cattle, which is higher than that found in our study, while the heritability of EMA (0.43) and MS (0.66) were similar in both studies. Conversely, heritabilities reported by Gordo et al. [19, 34] for BT (0.07; 0.21), CW (0.17; 0.21), and EMA (0.13; 0.28) in Nellore cattle are lower than those seen in our study. Heritabilities of carcass traits, except EMA, obtained in this study are different from those found by Bhuiyan et al. [35], who reported a heritability of 0.29 for BT, 0.51 for CW, 0.45 for EMA, and 0.22 for MS, in Hanwoo cattle slaughtered at ~30 months of age.

The results showed a noticeable increase in the accuracy of GEBVs when models combining pedigree and genomic information were applied to the single-step analysis (Fig 1A). Moreover, a clear increase in the accuracy of CW was noted when a multi-trait ssGBLUP model was utilized instead of a univariate ssGBLUP model (Fig 1A). This suggests that genomic data provide additional information for the estimation of breeding values, resulting in estimates that are more accurate than those obtained using only pedigree-based models. The accuracy increased from 19% to 39% on switching to ssGBLUP from BLUP models, for all traits. This is because genomic information can capture variation in Mendelian sampling, thereby increasing the accuracy of GEBVs [36, 37]. These results confirm that ssGBLUP yields a more accurate prediction than does BLUP, which is in concordance with findings of previous studies [17, 18, 20, 21, 24, 38–40]. In Japanese black cattle, Onogi et al. [18] demonstrated that on average, the ST-ssGBLUP model is 45% more accurate than ST-BLUP, in terms of the predictive ability, for carcass traits (CW, EMA, and MS). Similarly, Gordo et al. [19] reported that replacing **A** with the **H** matrix can improve the accuracy of carcass traits (BT, CW, and EMA) by approximately 17% in young Nellore males. The improved accuracies obtained in this study are greater than those reported by Lee et al. [20] for Hanwoo beef cattle. These authors stated that the ST-ssGBLUP model is more accurate than ST-BLUP by ~27% for CW, 24% for EMA, and 12% for MS. In contrast, the accuracy gain in our study after switching to ST-ssGBLUP from ST-BLUP was 42% for CW, 40% for EMA, and 32% for MS (Fig 1A). This is because we included a higher number of genotyped animals and phenotypic records than did Lee et al. [20], who analyzed 2,426 phenotypic records and 988 genotyped animals.

The use of multivariate models is known to provide more accurate breeding values than those obtained by single-trait models, as the information from genetically correlating traits can be utilized [25, 26, 37, 41–46]. Therefore, a multi-trait model may be better suited for traits with only a small number of phenotypes, but genetically correlated with traits having complete data. In the present study, the prediction for CW, EMA, and YW based on the multi-trait model was more accurate than that obtained using a single-trait model (Fig 1A), similar to findings of several other studies [25, 26, 41, 43].

Moreover, tThe genetic correlation existing between traits underlies the advantage of multi-trait models. In our study, the superiority of the multi-trait model (in terms of accuracy) over the single-trait model was more obvious for CW. The genetic correlation between CW and YW (0.76) was twice as strong as that between EMA and YW (0.34). These results also support the findings of Calus and Veerkamp [41], who pointed out that the inclusion of additional phenotypic data for an indicator trait (i.e., YW) increases the accuracy for the trait of interest (i.e., CW), when the genetic correlation is higher than 0.5. In contrast, the accuracy of EBV / GEBV for YW did not improve considerably on switching to a multi-trait model. This might be because the phenotypic dataset for YW was 2.6-fold larger than that for CW, similar to findings of Ismael et al. [43]. Their results revealed that ssGBLUP was more accurate (40%) than conventional BLUP models for “calving to the first insemination” (CFI) and “interval from calving to the first high activity” (CFHA) traits, in terms of reliability, and the best model for CFHA was MT-ssGBLUP. However, no difference was detected between single- and multi-trait models for CFI, because of the larger number of records available for CFI (1,472,313) than for CFHA (36,504) [43].

In the present study, analysis of a reduced dataset decreased the accuracy of YW breeding values in all models, compared with those obtained using the complete YW phenotypic data (Fig 1B). When additional phenotypic data on YW were eliminated from the training population, the phenotypic records of young bulls as relatives or sires of steers were lost, and the accuracy of EBVs / GEBVs declined for YW in BLUP or ssGBLUP. Moreover, the same phenotypic records for CW and YW resulted in the same number of genotyped animals (~921) for both traits within the training population which was less than the number of genotyped animals (~1233) for YW when complete data were used. The size of the training population has been shown to be one of the factors affecting the accuracy of GEBVs [47], which could further explain the decreased accuracy of GEBV for YW. In addition, a multi-trait model was more beneficial for YW when reduced data were used instead of the complete dataset (Fig 1A and 1B). On the other hand, the loss of accuracy decreased with the use of genomic data and/or a multi-trait model for YW with a missing part of data which might be due to greater heritability of CW (0.39) than YW (0.26), in line with the literature [25, 26, 42–45].

Our findings emphasize the importance of exploiting the high genetic correlation between traits by means of genomic multi-trait models. This approach is particularly relevant in practical breeding programs for traits such as CW, which are often not available for all animals under study.

## Conclusions

The results of this study indicate that the estimates of breeding values the single-step genomic methods are more accurate than those obtained using pedigree-based information for genetic evaluation of carcass traits and YW. Moreover, the multi-trait ssGBLUP model considerably increases the accuracy of breeding values for CW, a trait manifesting a high genetic correlation with YW, which is the most recorded trait. In general, the inclusion of genomic information in multi-trait methods may be a promising way to achieve acceptable accuracy of predictions. This approach is particularly relevant when phenotypic records are limited or missing for one of the two highly correlated traits. Furthermore, this approach could be suitable for evaluating genomic predictions, especially for young animals in the ongoing breeding programs on Hanwoo cattle.

## Supporting information

S1 Table Accuracy of breeding values obtained using the complete dataset of YW for each trait and fold number. (DOCX)

S2 Table Regression coefficients of the adjusted phenotype on the EBVs (bias) when a complete dataset of YW is used for each trait and fold number. (DOCX)

S3 Table Accuracy of breeding values obtained by means of a reduced dataset of YW for each trait and fold number (equal amounts of data for CW and YW). (DOCX)
