# Supplementary material for: Comparison of conventional BLUP and single-step genomic BLUP evaluations for yearling weight and carcass traits in Hanwoo beef cattle using single trait and multi-trait models
Source: PLoS One. 2019 Oct 14;14(10):e0223352. doi: 10.1371/journal.pone.0223352 (PMC6791548; doi:10.1371/journal.pone.0223352)
Supplement: S2 Table — (DOCX) [file pone.0223352.s002.docx]

**S2 Table**. Regression coefficients of the adjusted phenotype on the EBVs (bias) when a complete dataset is used for each trait and fold number

| Trait | Fold number | Animal number with record | Animal number with genotype and record within training population | Animal number with genotype and without record as validation population | Model | | | |
| --- | --- | --- | --- | --- | --- | --- | --- | --- |
|  |  |  |  |  | ST-BLUP | MT-BLUP | ST-ssGBLUP | MT-ssGBLUP |
| BT | 1 | 5,824 | 854 | 297 | 0.79 | 0.81 | 0.79 | 0.85 |
|  | 2 | 5,824 | 875 | 276 | 0.89 | 0.87 | 1.09 | 1.06 |
|  | 3 | 5,824 | 1006 | 145 | 0.16 | 0.05 | 0.14 | -0.01 |
|  | 4 | 5,824 | 844 | 307 | 0.78 | 0.76 | 0.93 | 0.90 |
|  | 5 | 5,824 | 1025 | 126 | 1.00 | 0.90 | 1.09 | 1.04 |
| CW | 1 | 5,824 | 854 | 297 | 0.76 | 0.87 | 1.22 | 1.20 |
|  | 2 | 5,824 | 875 | 276 | 1.35 | 1.31 | 1.56 | 1.35 |
|  | 3 | 5,824 | 1006 | 145 | 0.91 | 1.27 | 1.15 | 1.25 |
|  | 4 | 5,824 | 844 | 307 | 0.60 | 0.47 | 1.05 | 0.86 |
|  | 5 | 5,824 | 1025 | 126 | 1.81 | 1.41 | 1.60 | 1.31 |
| EMA | 1 | 5,821 | 854 | 297 | 0.81 | 0.89 | 1.19 | 1.23 |
|  | 2 | 5,821 | 875 | 276 | 1.02 | 1.03 | 1.31 | 1.25 |
|  | 3 | 5,821 | 1006 | 145 | 0.58 | 0.93 | 0.89 | 1.12 |
|  | 4 | 5,821 | 844 | 307 | 0.67 | 0.60 | 0.77 | 0.67 |
|  | 5 | 5,821 | 1025 | 126 | 1.13 | 1.13 | 1.10 | 1.02 |
| MS | 1 | 3,991 | 854 | 297 | 0.39 | 0.43 | 0.58 | 0.59 |
|  | 2 | 3,991 | 875 | 276 | 0.64 | 0.61 | 0.95 | 0.84 |
|  | 3 | 3,991 | 1006 | 145 | 0.67 | 0.73 | 0.92 | 0.98 |
|  | 4 | 3,991 | 844 | 307 | 0.78 | 0.73 | 0.75 | 0.69 |
|  | 5 | 3,991 | 1025 | 126 | 1.26 | 1.20 | 1.11 | 1.02 |
| YW | 1 | 15,279 | 1174 | 367 | 0.92 | 0.96 | 1.00 | 1.04 |
|  | 2 | 15,279 | 1184 | 357 | 1.02 | 1.06 | 1.27 | 1.28 |
|  | 3 | 15,279 | 1361 | 180 | 1.49 | 1.58 | 1.41 | 1.49 |
|  | 4 | 15,279 | 1074 | 467 | 1.21 | 1.26 | 1.21 | 1.18 |
|  | 5 | 15,279 | 1372 | 169 | 1.04 | 1.04 | 1.00 | 1.02 |
