# Supplementary material for: Comparison of conventional BLUP and single-step genomic BLUP evaluations for yearling weight and carcass traits in Hanwoo beef cattle using single trait and multi-trait models
Source: PLoS One. 2019 Oct 14;14(10):e0223352. doi: 10.1371/journal.pone.0223352 (PMC6791548; doi:10.1371/journal.pone.0223352)
Supplement: S3 Table — (DOCX) [file pone.0223352.s003.docx]

| Trait | Fold number | Animal number with record | Animal number with genotype and record within training population | Animal number with genotype and without record as validation population | Model | | | |
| --- | --- | --- | --- | --- | --- | --- | --- | --- |
|  |  |  |  |  | ST-BLUP | MT-BLUP | ST-ssGBLUP | MT-ssGBLUP |
| BT | 1 | 5,686 | 854 | 297 | 0.29 | 0.31 | 0.36 | 0.39 |
|  | 2 | 5,686 | 875 | 276 | 0.34 | 0.33 | 0.49 | 0.47 |
|  | 3 | 5,686 | 1006 | 145 | 0.04 | 0.02 | 0.05 | 0.00 |
|  | 4 | 5,686 | 844 | 307 | 0.30 | 0.29 | 0.40 | 0.38 |
|  | 5 | 5,686 | 1025 | 126 | 0.29 | 0.27 | 0.38 | 0.38 |
| CW | 1 | 5,687 | 854 | 297 | 0.26 | 0.25 | 0.45 | 0.45 |
|  | 2 | 5,687 | 875 | 276 | 0.49 | 0.49 | 0.64 | 0.64 |
|  | 3 | 5,687 | 1006 | 145 | 0.23 | 0.27 | 0.38 | 0.43 |
|  | 4 | 5,687 | 844 | 307 | 0.19 | 0.21 | 0.37 | 0.39 |
|  | 5 | 5,687 | 1025 | 126 | 0.49 | 0.47 | 0.53 | 0.52 |
| EMA | 1 | 5,684 | 854 | 297 | 0.27 | 0.28 | 0.46 | 0.46 |
|  | 2 | 5,684 | 875 | 276 | 0.42 | 0.42 | 0.57 | 0.56 |
|  | 3 | 5,684 | 1006 | 145 | 0.17 | 0.18 | 0.35 | 0.38 |
|  | 4 | 5,684 | 844 | 307 | 0.25 | 0.25 | 0.33 | 0.31 |
|  | 5 | 5,684 | 1025 | 126 | 0.37 | 0.37 | 0.40 | 0.39 |
| MS | 1 | 3,991 | 854 | 297 | 0.16 | 0.18 | 0.26 | 0.27 |
|  | 2 | 3,991 | 875 | 276 | 0.22 | 0.21 | 0.41 | 0.37 |
|  | 3 | 3,991 | 1006 | 145 | 0.20 | 0.23 | 0.32 | 0.35 |
|  | 4 | 3,991 | 844 | 307 | 0.35 | 0.33 | 0.39 | 0.37 |
|  | 5 | 3,991 | 1025 | 126 | 0.45 | 0.44 | 0.48 | 0.46 |
| YW | 1 | 5,687 | 854 | 367 | 0.33 | 0.34 | 0.46 | 0.48 |
|  | 2 | 5,687 | 875 | 357 | 0.40 | 0.45 | 0.55 | 0.61 |
|  | 3 | 5,687 | 1006 | 180 | 0.00 | 0.13 | 0.21 | 0.36 |
|  | 4 | 5,687 | 844 | 467 | 0.32 | 0.36 | 0.40 | 0.42 |
|  | 5 | 5,687 | 1025 | 169 | 0.55 | 0.54 | 0.49 | 0.52 |

**S3 Table**. Accuracy of breeding values obtained using a reduced dataset for each trait and fold number (equal amounts of data for CW and YW)
